# Structure-Forming Properties of *Pleurotus ostreatus*: A Promising Resource for Edible 3D Printing Applications

**DOI:** 10.3390/molecules30163350

**Published:** 2025-08-12

**Authors:** Alona Tiupova, Joanna Harasym

**Affiliations:** 1 Department of Biotechnology and Food Analysis, Wroclaw University of Economics and Business, Komandorska 118/120, 53-345 Wroclaw, Poland; alona.tiupova@ue.wroc.pl; 2 Adaptive Food Systems Accelerator-Science Centre, Wroclaw University of Economics and Business, 53-345 Wroclaw, Poland

**Keywords:** 3D food printing, *Pleurotus ostreatus*, β-glucans, rheological properties

## Abstract

Approximately 20–30% of cultivated oyster mushrooms (*Pleurotus ostreatus*) are classified as low grade due to morphological and visual imperfections or mechanical damage, representing significant waste in mushroom production systems. This review examines the structural and biochemical properties of *P. ostreatus*, particularly focusing on cell wall components including chitin, β-glucans, and mannogalactans, which provide crucial rheological characteristics for 3D printing. The literature results demonstrate that these natural polysaccharides contribute essential viscosity, water-binding capacity, and mechanical stability required for printable edible inks. Notably, the mushroom stipe contains significantly higher concentrations of glucans compared to the cap, with 57% more α-glucans and 33% more β-glucans. The unique combination of rigidity from chitin, elasticity from β-glucans, and water retention capabilities creates printable structures that maintain shape fidelity while delivering nutritional benefits. This approach addresses dual challenges in sustainable food systems by reducing agricultural waste streams while advancing eco-friendly food innovation. The integration of mushroom-derived biomaterials into 3D printing technologies offers a promising pathway toward developing nutrient-rich, functional foods within a regenerative production model.

## 1. Introduction

The global shift toward sustainable food systems has intensified the interest in circular economy principles and innovative technologies capable of minimizing waste while enhancing resource efficiency [1]. The circular economy model offers a promising framework to transform traditional linear food production into regenerative systems that minimize waste and optimize resource use.

While commercial cultivation of *Pleurotus ostreatus* (oyster mushrooms) generates a significant fraction of underutilized biomass rich in bioactive compounds, approximately 20–30% of cultivated fruiting bodies are classified as “low-grade” due to morphological anomalies, dimensional variations, or cellular disruption, rendering them unsuitable for direct consumption [2]. The production of 450 g of marketable *P. ostreatus* fruiting bodies yields approximately 18 g of underutilized biomass [3].

This discarded fungal material contains valuable β-glucans, glucomannans, chitin, ergosterol, ergothioneine, phenolic compounds, and numerous enzymes that are typically lost during conventional disposal methods [4]. Typical management approaches fail to recover these high-value bioactive molecules and compromise their structural integrity, which is critical for their functional properties.

Concurrently, additive manufacturing in food processing (3D food printing) has emerged as a technique for creating customized, structured food matrices with controlled release of bioactive compounds [5]. This technology offers a promising approach for preserving the biological activity and molecular structure of mushroom-derived compounds by incorporating them into printable edible bioinks with controlled temperature and shear conditions. However, a significant challenge lies in maintaining the molecular weight and tertiary structure of fungal polysaccharides during processing. Current formulations predominantly subject these compounds to depolymerization through high-temperature processing or chemical modifications, significantly diminishing their immunomodulatory activity and rheological functionality [6]. The development of appropriate bioink formulations presents multiple biochemical challenges, including maintaining polysaccharide chain length, preserving ergosterol content, and ensuring the stability of heat-sensitive phenolic compounds during extrusion [7].

Low-grade oyster mushrooms, rich in dietary fibers, β-glucans, and texturally cohesive microfibrillar structures, present an unexploited resource for developing nutrient-rich, rheologically stable edible inks for 3D printing [8,9]. Their natural polysaccharide and fiber content can contribute to the viscosity and gelation properties required for stable and precise printing. Utilizing these mushrooms as a base for edible inks not only addresses waste management issues but also offers economic benefits by reducing disposal costs and creating value-added products. Environmentally, this approach contributes to waste reduction and a lower carbon footprint, while functionally, it enhances the nutritional profile of printed foods.

This intersection of food waste valorization and advanced manufacturing underscores the potential of 3D food printing to convert mushroom by-products into high-value applications. By repurposing low-grade oyster mushroom into printable inks, this approach addresses dual imperatives: mitigating waste streams in agriculture and advancing eco-friendly food innovation. Identifying suitable biomaterials for 3D food printing is crucial, as factors such as viscosity, shear behavior, and stability directly impact printability and final product quality. Mushroom-derived polysaccharides have been explored as sustainable functional materials in this context, offering promising results [9,10]. Such efforts align with broader initiatives to identify biomaterials that balance printability, nutritional integrity, and environmental sustainability—a critical step toward scalable, circular food systems [11].

The present review aims to bridge the gap between sustainable resource utilization and technological innovation by exploring the potential of low-grade *Pleurotus ostreatus* as a functional material for edible 3D printing applications. By synthesizing current scientific knowledge on the structural, rheological, and nutritional properties of oyster mushrooms, this work demonstrates how underutilized fungal biomass can be transformed into high-value, customizable food products. The integration of *P. ostreatus* into 3D food printing aligns with circular economy principles, offering an eco-friendly solution to food waste while advancing the development of nutritionally enriched, structurally stable, and consumer-acceptable functional foods. This approach supports broader goals of food system resilience, resource efficiency, and sustainable innovation in the agri-food sector.

## 2. Structural and Textural Properties of Oyster Mushrooms

### 2.1. Cell Wall Composition

The cell wall of *P. ostreatus* is a complex structure primarily composed of chitin, β-glucans, and other polysaccharides like lentinan and mannogalactans (Table 1). These components provide rigidity, elasticity, and water-binding capacity, making the mushroom both structurally robust and adaptable [12,13]. These same properties play a crucial role in 3D printing, where the material’s ability to retain moisture, maintain its shape, and provide mechanical stability determines the quality and functionality of printed structures [9].

The analysis of different production strains of *P. ostreatus* reveals significant variation in glucan distribution between the stipe and cap. The stipe consistently contains higher levels of total glucans, α-glucans, and β-glucans compared to the cap. Specifically, α-glucans are 57% more concentrated in the stipe, while β-glucans are 33% higher than in the cap. The findings highlight the stipe as the primary glucan-rich region, which could have implications for the valorization of *P. ostreatus* [4,9]. The mechanical strength of chitin arises from its highly ordered crystalline structure, where β-(1,4)-linked N-acetylglucosamine chains form extensive hydrogen bonding networks and van der Waals interactions, creating a rigid three-dimensional framework that can withstand mechanical stress during extrusion and layer deposition. In 3D printing applications, chitin’s fibrous architecture acts as a natural reinforcement matrix, where individual polymer chains align under shear forces to distribute mechanical loads throughout the printed structure, thereby preventing deformation and maintaining the dimensional stability of the extruded material.

The ability of oyster mushroom polysaccharides to retain water plays a particularly important role in ensuring printability. Water retention prevents cracking and drying during and after printing, maintaining the intended texture and stability of the printed object. The combination of chitin’s rigidity, β-glucans’ elasticity, and the water-binding properties of additional polysaccharides makes *P. ostreatus* an excellent candidate for applications in food printing, biopolymer development, and biodegradable packaging [18,19]. Its natural biodegradability and renewability further position it as a sustainable alternative to synthetic polymers, expanding its potential beyond food into biomedical and environmental applications [9,20].

Thanks to those unique structural properties, *P. ostreatus* emerges as a promising material for 3D printing, offering a natural, adaptable, and sustainable approach to producing printed structures with tailored mechanical and functional characteristics [21].

### 2.2. Mechanical Behavior

Mycelium-based materials, particularly those derived from *P. ostreatus*, exhibit unique mechanical properties that make them highly suitable for 3D printing [9,22]. A defining characteristic of these materials is their natural shear-thinning behavior, a non-Newtonian property where viscosity decreases under applied stress. This rheological trait is particularly advantageous for extrusion-based additive manufacturing, as it enables smooth flow through a nozzle while maintaining structural integrity post-deposition [23,24]. The shear-thinning effect arises from the dynamic interactions of mycelial biopolymers, primarily β-glucans and chitin, which reorganize under shear forces to facilitate extrusion while rapidly recovering structural stability after deposition [25,26]. The mechanical strength of chitin arises from its highly ordered crystalline structure, where β-(1,4)-linked N-acetylglucosamine chains form extensive hydrogen bonding networks and van der Waals interactions, creating a rigid three-dimensional framework that can withstand mechanical stress during extrusion and layer deposition. In 3D printing applications, chitin’s fibrous architecture acts as a natural reinforcement matrix, where individual polymer chains align under shear forces to distribute mechanical loads throughout the printed structure, thereby preventing deformation and maintaining dimensional stability of the extruded material.

Rheological properties of *P. ostreatus* pastes or related biomass inks are typically characterized using a controlled-temperature rotational rheometer equipped with cone-plate or parallel-plate geometry. A steady shear rate sweep (0.01–100 s^−1^) is conducted to capture the material’s shear-thinning behavior, with data fitted to the power law model (τ = K·γ^n^). A flow behavior index (n) significantly below 1 confirms pseudoplastic flow. Yield stress is determined from stress ramp or crossover in steady shear. Small-amplitude oscillatory shear tests across a frequency range provide the storage modulus (G′), loss modulus (G″), and loss tangent (tan δ); printable formulations show G′ > G″ and tan δ < 1 [19,21]. These rheological studies confirm that oyster mushroom mycelium pastes exhibit yield stress behavior followed by pseudoplastic flow. This allows precise control over material deposition, enhancing print resolution and geometric fidelity. Additionally, the viscoelastic properties of mycelium composites can be fine-tuned through cultivation conditions, enabling tailored mechanical responses suited to diverse fabrication needs.

#### 2.2.1. Texture-Forming Properties

Oyster mushroom mycelium exhibits intrinsic gelation properties due to its high glucan and chitin content. The β-1,3/1,6-glucans within the cell walls establish a three-dimensional network through hydrogen bonding, promoting gel formation [27,28]. This gelation mechanism enables the material to form cohesive and self-supporting structures essential for stable additive manufacturing.

**Figure 1:**
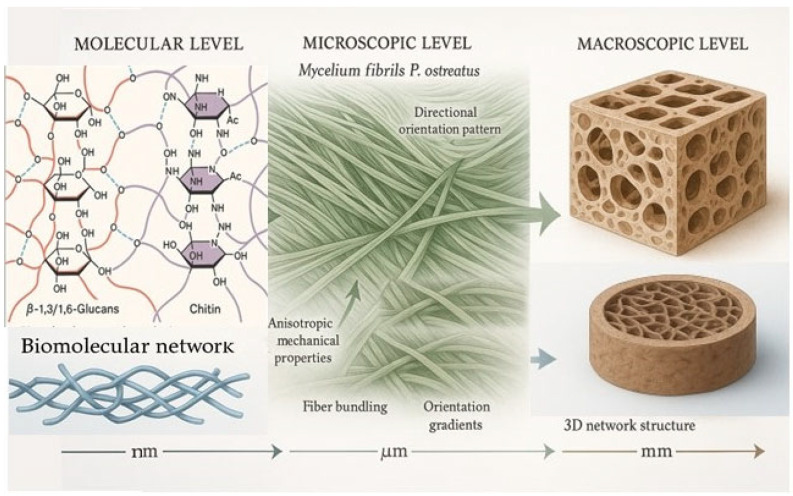
Hierarchical structure of mycelium fibers from *P. ostreatus.* The molecular level shows the fundamental β-glucan and chitin components that form biomolecular networks through hydrogen bonding [25,26,27]. The microscopic level shows mycelium fibrils with directional orientation during extrusion processes, contributing to anisotropic mechanical properties [36,37,38]. The macroscopic level shows the 3D network structure in printed composites that determines the material’s functional behavior [22,39,40].

The water retention ability of mycelium-based materials significantly impacts their rheological behavior and post-processing characteristics [29,30]. Oyster mushroom-derived polysaccharides bind water molecules through multiple hydrogen bonding sites, forming hydrated networks that influence printability and drying kinetics [31,32]. *P. ostreatus* powder is characterized by a high water-holding capacity (13.46 g/g). β-Glucan is a soluble dietary fiber composed of structural polysaccharides formed by D-glucose monomers, exhibiting beneficial health properties. As a water-soluble polymer, β-glucan demonstrates a strong ability to bind water. Dietary fibers, including β-glucan present in oyster mushrooms, contribute to water-binding capacity [33]. Proper hydration control is crucial for preventing brittleness, maintaining flexibility, and ensuring dimensional stability in printed structures [34,35]. The multi-scale organization of mycelium fibers across three structural levels is shown on Figure 1.

The hierarchical fibrous structure of mycelium exhibits anisotropic mechanical properties, which are particularly evident in printed composites. Controlled fiber orientation enables precise modeling of mechanical characteristics, optimizing the strength and elasticity of the resulting components [36]. During extrusion, transient forces contribute to the formation of competitive structures, enhancing tensile strength along the printing direction [37]. However, a reduction in movement speed leads to a more random fiber distribution, which, in turn, affects the texture and elasticity of the material.

The mechanical properties of mycelium depend on processing conditions. During the extrusion of protein-based material with added pigment, a decrease in retention capacity is observed, leading to an increased expansion coefficient of the extrudate and its propagation along the fibers [37]. Additional studies conducted on *P. eryngii* fruiting bodies have revealed the presence of curls and irregular formations within the fibrous structure [38], which affect the mechanical strength and physicochemical bending properties of the matrix.

The rheological behavior of mycelium-based materials, such as those derived from *P. ostreatus*, is significantly influenced by processing conditions, including temperature, pH, and ionic concentration. Research on *Beauveria bassiana* fermentation broths demonstrated that viscosity increased significantly at higher stirring speeds, which correlates with temperature variations during processing [39]. Ionic concentration also affects the rheological behavior of biopolymer solutions. Research on microbial extracellular polysaccharides has shown that viscosity retention rates change with varying salinity levels [35,41,42]. While direct studies on mycelium-based materials are limited, it is reasonable to infer that ionic strength adjustments could similarly influence their flow behavior, necessitating careful control of ionic conditions during processing.

The particle size distribution within mycelium-based composites, particularly those utilizing *P. ostreatus*, significantly influences both their printability and mechanical properties. Studies have demonstrated that incorporating smaller particles enhances the uniformity and resolution of printed structures, while larger particles contribute to increased mechanical strength [22,43]. Other researchers found that composites with smaller particles exhibited a soft elastic response at small strains, mimicking pure mycelium behavior, and demonstrated marked stiffening at larger strains due to particle densification. Conversely, composites with larger particles showed increased stiffness and strength, highlighting the role of particle size in tailoring mechanical properties [44,45].

#### 2.2.2. Structure–Function Relationships

The structural integrity and functional performance of *P. ostreatus* depend on its cell wall composition and dynamic interactions between biomolecules. The mycelium exhibits shear-thinning behavior, where viscosity decreases under applied stress, making it suitable for extrusion-based 3D printing [27,28]. Oscillatory rheological measurements reveal a frequency-dependent viscoelastic response, with the storage modulus (G′) and loss modulus (G″) exhibiting nonlinear relationships under varying mechanical stresses. The results suggest that the alignment of β-glucan and chitin fibrils under shear forces contributes to these properties [36].

Heating induces gelation due to the denaturation of proteins and reorganization of polysaccharide networks. Thermal transitions between 20 and 45 °C induce molecular rearrangements, particularly affecting protein conformation and polysaccharide network configurations. At temperatures above 60 °C, β-glucans form thermally stable hydrogels, while excessive heat (>90 °C) degrades chitin, reducing mechanical strength [18,41].

The cell wall’s stability is pH-dependent; acidic conditions (pH 3–5) enhance hydrogen bonding between polysaccharides, increasing rigidity, while alkaline conditions (pH > 8) disrupt these bonds, leading to the solubilization of β-glucans [46,47].

## 3. Valorization of Low-Grade Oyster Mushroom—Structural, Functional, and Economic Perspectives

**Figure 2:**
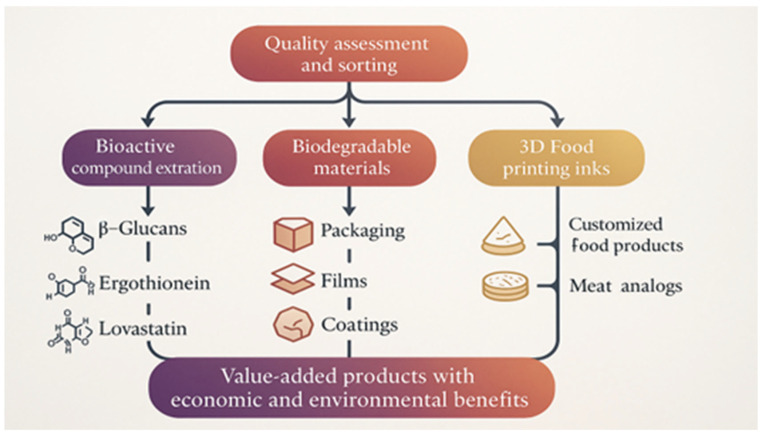
Valorization process for low-grade *P. ostreatus* [48,49].

Low-grade *P*. *ostreatus* are those that deviate from commercial standards due to irregular growth patterns, aesthetic imperfections, or structural asymmetries. However, they maintain their biochemical integrity, making them suitable for secondary applications [4]. The valorization scheme for low-grade *P*. *ostreatus* is shown in Figure 2.

Studies show that deviations of ±15–25% from standard dimensions do not significantly impact the biochemical composition of mushrooms [48]. Modern geometric morphometric techniques assess variations in cap curvature and gill alignment, providing insights into genetic or environmental influences on growth. High-resolution imaging, such as micro-computed tomography (µCT), is used to evaluate internal density fluctuations, which may impact texture and bioavailability in downstream applications [48,49].

Analyses assessing environmental impact show a reduction in carbon emissions by up to 68% when compared to conventional disposal methods such as incineration or landfilling. Resource optimization and efficient processing techniques reduce water and energy consumption. Waste stream minimization, integrating mushroom waste into biorefinery systems, prevents organic waste accumulation [4,50].

Industrial applications and the market viability of oyster mushrooms may lie in bioprocessing; for example, enzymatic hydrolysis and supercritical fluid extraction increase the efficiency of extraction of bioactive compounds. The extraction of proteins and polysaccharides and alternative protein sources are also used [47,49,50]. But all these methods still have a relatively high percentage of waste, in contrast to bioinks for 3D printing, which in turn can increase the cost effectiveness. Techno-economic assessments show that the use of low-grade mushrooms can reduce raw material costs by 70–75% [51,52].

## 4. Functional Advantages of Pleurotus Ostreatus in 3D Printing

The field of additive manufacturing has increasingly explored alternative biomaterials, expanding research into non-traditional substrates with specific physicochemical properties. *P. ostreatus* (oyster mushroom) represents a significant advancement in biomaterial science, combining structural flexibility, rheological stability, and nutritional complexity that makes it particularly valuable for 3D food printing and biopolymer applications. Its functionality as a sustainable printing material is directly derived from its rheological characteristics, molecular structure, and performance after processing.

Research shows that mushroom-derived materials, especially those from *P. ostreatus*, demonstrate exceptional pseudoplastic behavior. Their complex polysaccharide composition, primarily chitin and β-glucans, creates a shear-thinning rheological profile that is essential for extrusion-based printing techniques [12,26]. When examined under varying stress conditions, mushroom paste displays classic non-Newtonian fluid dynamics: viscosity decreases as shear stress increases, flow properties improve during extrusion, and the material rapidly recovers its structure after deformation [53]. This combination of properties addresses several challenges in biofabrication. The shear-thinning behavior facilitates smooth extrusion through narrow printing nozzles, while the rapid structural recovery maintains print geometry without collapsing. Additionally, the natural composition offers nutritional benefits without requiring synthetic additives, positioning *P. ostreatus* as a promising candidate for sustainable manufacturing solutions. These properties facilitate precise material deposition and immediate shape retention, fundamental requirements for sophisticated 3D printing methodologies. Studies indicate that modifying hydration levels and particle size distribution further optimizes these properties for controlled layer-by-layer deposition [24,54].

**Figure 3:**
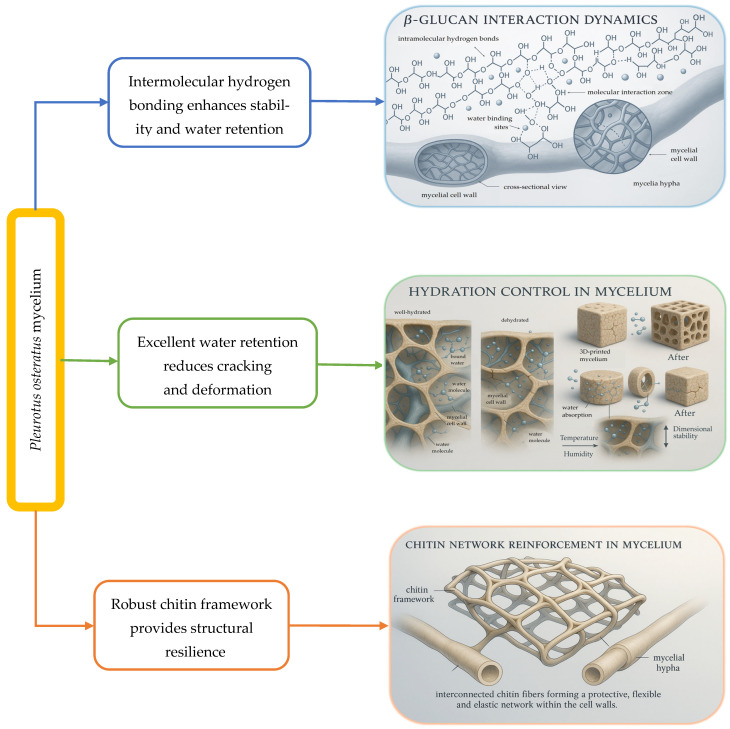
Natural stabilization mechanisms in *P. ostreatus* mycelium structure. (1) Chitin network reinforcement: the robust chitin framework contributes structural resilience, minimizing deformation during printing and subsequent thermal processing [24]. (2) β-Glucan interaction dynamics: these complex polysaccharides generate intermolecular hydrogen bonding (depicted as dashed lines between chains), enhancing mechanical stability and water retention capabilities [53]. (3) Hydration control: experimental observations indicate that *P. ostreatus* exhibits superior water retention compared to traditional organic substrates, reducing cracking and deformation during the dehydration processes [24,53].

The intrinsic molecular architecture of *P. ostreatus* provides natural stabilization through several key mechanisms (Figure 3).

The presence of chitin and β-glucans in *P. ostreatus* contributes to the ink’s viscosity and stability. Wang et al. (2025) demonstrated that the incorporation of *Pleurotus ostreatus* mycelium into 3D printing formulations for dysphagia-friendly foods can improve material processability [19]. Similarly, Mohamad Mazlan et al. (2020) reported that combining mushroom paste with plant-derived hydrocolloids, such as soy protein, enhances viscosity control and printing resolution during extrusion-based food printing [37]. The integration of *P. ostreatus* into 3D printing materials improves the final product integrity, yielding structures with enhanced mechanical stability and controlled porosity. Studies have demonstrated that optimized formulations prevent layer delamination and allow for controlled texture engineering [55].

Santhapur et al. (2024) developed hybrid 3D-printed vegan meat analogs using whey protein and mushroom-derived hydrogels, including *P. ostreatus*, and reported enhanced umami amino acid content, resulting in a more savory flavor profile [35]. Mohamad Mazlan et al. (2020) observed that the addition of oyster mushroom powder reduced mechanical properties such as hardness, stiffness, springiness, and chewiness, yielding a juicier and more palatable product [37]. Godschalk-Broers et al. (2022) further supported these findings by linking mushroom incorporation to improved sensory characteristics and consumer acceptability in meat analogs [56]. Zhang et al. (2024) demonstrated that mushroom-enriched formulations exhibited better printability and allowed for material recycling without compromising the structural fidelity of the printed product [57]. Complementary insights from Afzal et al. (2018) on the rheological behavior of biopolymer-based hydrogels also reinforce the potential of such composites for extrusion-based applications [40].

Lin et al. (2024) investigated the incorporation of living *Pleurotus ostreatus* mycelium into a food-grade hydrogel matrix for 3D bioprinting applications [9]. Their formulation included malt extract to promote mycelial viability, carboxymethylcellulose and cornstarch to modulate rheological properties, and agar to enhance structural integrity. The printed constructs exhibited continued mycelial growth post-printing, demonstrating potential for the development of engineered living materials applicable to both food and sustainable packaging sectors [9,35].

In a related study, Keerthana et al. (2020) developed fiber-enriched 3D-printed snacks using wheat flour combined with button mushroom powder [10]. Their results showed that formulations containing up to 20% mushroom powder maintained good printing precision and structural stability, while significantly enhancing the dietary fiber content of the final product. Although the study utilized Agaricus bisporus, the findings suggest that *P. ostreatus* powder may offer similar functional benefits in the design of nutritionally enriched, printable snack matrices [10].

The key technological parameters applied in 3D printing with *Pleurotus mycelium*-based materials, along with corresponding literature sources, are systematically summarized in Table 2.

During thermal processing, *P. ostreatus*-based substrates undergo Maillard reactions, contributing to enhanced structural cross-linking. Improved mechanical strength and nuanced flavor development in food-grade applications not only improve the mechanical robustness of printed structures but also introduce sensory advantages, making *P. ostreatus* an attractive material for functional food printing [61,62]. The thermal stability of mushroom-derived materials supports their applicability in high-temperature processing environments without significant structural degradation.

Oyster mushroom mycelium-based materials are environmentally friendly and biodegradable, making them attractive as a replacement for traditional materials such as polystyrene, which are energy-intensive to manufacture [9,63]. Other waste materials such as coffee grounds and agricultural waste are used to create mycelium-based materials, which helps reduce waste and increase sustainability [25,63,64]. Mycelium composites have competitive mechanical properties such as high compressive strength, making them suitable for use in construction and packaging [21,25,63]. Objects created from such materials can self-heal and be used to bond components, which increases their functionality [62]. Three-dimensional printing using mycelium can be carried out under non-sterile conditions, which simplifies the manufacturing process [9,64].

## 5. Edible Applications of Pleurotus Ostreatus Mycelium

The macronutrient profile of *P. ostreatus* is characterized by a high protein content (15–25% dry weight), complex carbohydrates, and minimal fats, making it an excellent low-calorie food source. Studies have demonstrated that oyster mushrooms contain all essential amino acids, with particularly high levels of lysine and leucine, which are often limited in plant-based diets [65,66].

The cell wall of *P. ostreatus* consists primarily of β-glucans and chitin, contributing to its high fiber content. β-Glucans have been extensively studied for their prebiotic properties, promoting gut microbiota health and improving overall digestion [18,27].

Numerous bioactive compounds present in *P. ostreatus* reflect its health-promoting effects, contributing strong antioxidant activity and neutralizing free radicals [65,67]. Among these, several naturally occurring antioxidants have shown promising neuroprotective and anti-inflammatory potential [48,68]. In addition, polysaccharides such as lentinan and pleuran have demonstrated immunomodulatory and anti-cancer activities by enhancing various aspects of the immune response [69,70].

## 6. Dietary Flexibility and Allergen-Free Attributes

### 6.1. Suitability for Various Dietary Patterns

As a plant-based, naturally gluten-free and minimally processed ingredient, P. ostreatus fits into a variety of dietary lifestyles, thanks to its composition, which is presented in the Table 3.

Due to their high protein content and complete amino acid profile, *P. ostreatus* mushrooms represent a valuable dietary protein source, especially for vegetarian and vegan consumers. Godschalk-Broers et al. (2022) and Zhang et al. (2024) demonstrated that mushroom-based formulations offer a protein-rich alternative to meat analogs, while also contributing umami flavor without relying on animal-derived components [56,57]. Gluten-free diets, which exclude gluten-containing grains such as wheat, rye, and barley, are essential for individuals with celiac disease or non-celiac gluten sensitivity [72,73]. Similarly, low-fat and heart-healthy dietary patterns, characterized by minimal intake of saturated fats, have been associated with improved cardiovascular outcomes, including reductions in LDL cholesterol levels [74].

### 6.2. Flavor Neutrality and Versatility

*P. ostreatus* has been identified as a promising candidate for edible 3D printing ink, which can be used to create innovative and nutritious food products. The mushroom’s ability to maintain stable intermolecular structures when mixed with other ingredients like fats highlights its potential in culinary innovation [24]. In a related application, Özünlü and Ergezer (2021) incorporated dried *P. ostreatus* into beef salami formulations, reporting enhanced nutritional profiles and extended shelf-life [74]. Their study demonstrated that supplementation with 1–5% oyster mushroom powder reduced lipid and protein oxidation during storage, without negatively impacting flavor or overall sensory acceptability [74].

This mushroom has been used in the development of functional foods, such as cookies, where it enhances antioxidant capacity and protein bioavailability [75]. *P. ostreatus* is rich in proteins, carbohydrates, and essential micronutrients, including minerals and phenolic compounds, which contribute to its health benefits and culinary appeal [76,77].

The edible applications of *P. ostreatus* extend beyond traditional culinary uses, presenting opportunities in functional foods, dietary supplementation, and sustainable food innovation. Acute toxicity assessments of *P. ostreatus* powder have demonstrated its safety for consumption. Oral administration in animal models at doses up to 2000 mg/kg did not result in adverse effects, supporting its use as a nutraceutical and functional food component [76].

## 7. Technical Problems and Standardization Strategies

The utilization of *P. ostreatus* as a raw material for 3D food printing presents both promising opportunities and significant challenges. While its structural and nutritional properties make it a suitable candidate for bioink formulations, several technical, regulatory, and consumer-related barriers must be addressed for its successful integration into food technology.

### 7.1. Variability in Raw Material Consistency

One of the primary obstacles in utilizing *P. ostreatus* for 3D printing is the inherent variability in its composition, which affects rheological properties and printability. The fungal biomass is influenced by substrate composition, growth conditions, and post-harvest processing methods, leading to inconsistencies in moisture content, fiber concentration, and the protein-to-polysaccharide ratio [78]. This variability impacts extrusion behavior, layer adhesion, and final texture stability in printed structures.

To mitigate inconsistencies, a controlled processing approach is essential. Standardized drying and milling techniques can ensure uniform particle size distribution and moisture content, leading to improved batch-to-batch reproducibility. Freeze-drying or convective drying followed by fine milling has been suggested as an effective strategy to maintain the structural integrity of mushroom-derived materials while enhancing flow properties for extrusion-based applications [79]. Additionally, blending with hydrocolloids or other structuring agents, such as alginate or carrageenan, can further stabilize the rheological behavior of *P. ostreatus*-based bioinks [23,54].

### 7.2. Microbial Contamination Risk

The high moisture content and nutrient-rich nature of *P. ostreatus* make it susceptible to microbial growth, posing food safety risks during processing and storage. Contamination with spoilage bacteria and fungi can alter sensory attributes and compromise the functional properties of bioink formulations [9]. Thermal and non-thermal pasteurization techniques can effectively reduce microbial load while preserving bioactive compounds. Mild heat treatments (e.g., 70–80 °C for short durations) have been shown to maintain the integrity of polysaccharides and proteins in fungal materials [21]. Alternative methods such as high-pressure processing and pulsed electric field treatment offer non-thermal preservation solutions that extend shelf life without compromising textural and nutritional properties [80]. The integration of antimicrobial packaging or natural preservatives (e.g., plant-based phenolics) can further enhance microbial stability in *P. ostreatus* bioink formulations.

### 7.3. Educating Consumers on Safety and Benefits of Upcycled Ingredients

Despite the sustainability and nutritional advantages of *P. ostreatus*-derived food products, consumer acceptance remains a significant challenge. The perception of upcycled food ingredients, particularly those derived from fungal biomass, may be associated with concerns over safety, taste, and unfamiliarity with the concept of 3D-printed foods [81]. To enhance consumer confidence, clear labeling and education regarding the benefits of mushroom-based bioinks are crucial. Public awareness campaigns highlighting sustainability aspects, such as the valorization of by-streams and reduced environmental footprint, can improve market reception. Scientific validation through sensory analysis and consumer studies can further support claims related to taste, texture, and overall acceptance [82]. Additionally, collaborations with culinary professionals and food influencers can help normalize 3D-printed foods containing *P. ostreatus* as a novel and nutritious alternative.

### 7.4. Compliance with Food Safety Standards

The introduction of *P. ostreatus*-based bioinks into the food industry necessitates adherence to food safety regulations, which vary by region. The classification of fungal-derived bioinks as novel food ingredients requires thorough risk assessment and documentation to ensure compliance with standards set by regulatory bodies such as the European Food Safety Authority (EFSA) and the U.S. Food and Drug Administration (FDA). A structured regulatory approach should include the analysis of potential allergens, antinutritional factors, and contaminants [42], detailed compositional analysis demonstrating the safety and health benefits of *P. ostreatus*-derived products, stability and shelf-life testing ensuring that bioink formulations remain stable under recommended storage conditions without microbial spoilage or degradation of functional properties, and the implementation of standardized production methodologies aligned with Hazard Analysis and Critical Control Points (HACCP) guidelines [78]. By actively engaging with regulatory agencies and industry stakeholders, the integration of *P. ostreatus* into 3D food printing can be streamlined, ensuring both safety and market acceptance.

## 8. Future Directions

Achieving consistent printability while preserving the nutritional and textural properties of *P. ostreatus*-based inks necessitates a comprehensive study of them. The rheological properties of edible inks are highly temperature-dependent. Optimizing the extrusion temperature can prevent the degradation of bioactive compounds while maintaining proper flow dynamics [24,83].

The mechanical behavior of ink under shear stress during extrusion affects both structural integrity and surface resolution. Innovations in nozzle geometry and extrusion pressure regulation can enhance layer adhesion and minimize print defects [84,85]. Evaluating drying, freeze-drying, or controlled dehydration methods can improve product longevity while maintaining edibility and texture [54,86]. Future research should prioritize in-depth mechanistic studies to elucidate the precise structure–function relationships between individual cell wall components (chitin, β-glucans, and mannogalactans) and their synergistic interactions, employing advanced characterization techniques such as molecular dynamics simulations, X-ray crystallography, and real-time rheological analysis under printing conditions to establish quantitative correlations between molecular structure and functional performance in 3D printing applications.

To enhance nutritional diversity and structural robustness, hybrid inks incorporating *P. ostreatus* with complementary bio-based materials warrant exploration. Combining *P. ostreatus* with microalgae (e.g., *Spirulina platensis*) can enhance protein and micronutrient content while offering improved textural and coloration properties [87]. Integrating mycoprotein with insect-derived proteins (e.g., *Tenebrio molitor* larvae) may optimize amino acid profiles and fortify the mechanical resilience of 3D-printed structures [58]. Synergistic interactions between fungal biomass and plant-based polysaccharides (e.g., pectin and alginate) could modulate viscosity and extrusion behavior, leading to improved print fidelity [23,54].

Edible 3D printing has demonstrated potential in producing customized nutritional products, personalized dietary solutions, and aesthetically complex food designs [24,88,89]. The incorporation of *P. ostreatus* aligns with efforts to enhance food sustainability while leveraging fungal biomass as a functional ingredient. Mycoprotein-based structures mimicking traditional meat textures can expand the scope of plant-based diets while reducing reliance on resource-intensive animal agriculture [54,56].

To facilitate commercial adoption, a techno-economic assessment is essential. The relative costs of raw material acquisition, processing, and formulation must be weighed against conventional food production methodologies. Evaluating consumer perception, sensory acceptance, and regulatory compliance will determine the commercial feasibility of mushroom-based 3D food products. Developing sustainable supply chains from agricultural by-streams to edible ink formulations could enhance economic competitiveness while promoting circular bioeconomy models [64].

Despite significant advancements, several research gaps remain. Investigating the impact of extrusion, drying, and storage on bioactive compound stability is critical. Ensuring food safety through the integration of natural antimicrobials or controlled fermentation strategies warrants further study. Standardizing labeling, safety regulations, and consumer education will facilitate broader market integration.

## 9. Conclusions

The hierarchical fibrous structure of *Pleurotus ostreatus* exhibits anisotropic mechanical properties, making it a viable candidate for edible 3D printing inks. Controlled fiber orientation allows for the precise modeling of mechanical characteristics, optimizing the strength and elasticity of printed components. During extrusion, transient forces contribute to the formation of competitive structures, enhancing tensile strength along the printing direction. However, adjusting printing parameters, such as reducing movement speed, leads to a more randomized fiber distribution, which influences the texture and elasticity of the final product.

The mechanical properties of *Pleurotus ostreatus* are highly dependent on processing conditions. During the extrusion of mycelium-based inks with added pigments, variations in retention capacity influence the expansion coefficient and the overall printability of the material. This adaptability ensures that *P. ostreatus* can maintain structural integrity during layer-by-layer deposition, making it a suitable material for 3D food printing applications.

To optimize *Pleurotus ostreatus* for edible 3D printing, further refinement of extrusion parameters is necessary to balance printability and texture. Investigating the impact of pre-processing treatments, such as hydration control and structural modification, could enhance its performance in food printing applications. Additionally, integrating *P. ostreatus* into existing food supply chains as a functional and sustainable ingredient could facilitate widespread adoption in the culinary and food technology industries.

## Figures and Tables

**Table 1:** Cell wall composition of *P. ostreatus* and contribution to 3D printing.

Component	Structure	Functional Properties (Quantitative)	Contribution to 3D Printing	Ref.
Chitin	β-(1,4)-linked polymer of N-acetylglucosamine	- Tensile strength (UTS): ~32–36 MPa (in biocomposites) - Young’s modulus: ~1.1 GPa - In foamed structures: modulus 3–175 MPa, UTS 0.15–6.5 MPa	- High mechanical strength allows structural stability - Stiffness (~1 GPa) aids shape retention - Lightweight porous structure	[13,14,15]
β-glucans	β-(1,3)- and β-(1,6)-D-glucans	- Elastic modulus: ~0.9 MPa (in 1% β-glucan starch patch) - Tensile strength: ~0.92 MPa - Elongation: ~50%	- Provides elasticity and flexibility - Enhances moisture retention (up to 60%) - Improves printable texture and formability	[16,17]
Mannogalactans	Complex polysaccharides	- Water-holding capacity increase: +10–15% (vs. control) - Elastic modulus (G′): ~16.7 kPa	- Improves water retention and flexibility - Enhances print accuracy (deviation < 5%), retains shape for up to 5 h	[12,18]
Proteins	Various	- With 6% SPI: G′ increased by ~30%, significant water-holding capacity improvement (*p* < 0.05) - Print accuracy: ~97.9%, stability ~98.2%	- Enhances layer adhesion and structural integrity - Increases moisture retention and form stability in food inks	[12,18]

**Table 2:** Process parameters and applications of mushroom-based 3D food printing.

Composition	Printing Parameters	Printed Product/Observations	Ref.
*P. ostreatus* hydrogel + CMC + agar + starch	Extrusion mode: Syringe Nozzle: Not specified Temperature: Room temperature Pressure/Speed: Not reported Layer height: Not reported	Printable mycelium-rich food-grade hydrogel; stable form; biocompatible and edible; no sterile environment required	[58]
*P. ostreatus* paste (rheology-focused)	Extrusion mode: Pneumatic Nozzle/Pressure/Speed: Not reported Rheology: Studied; flow affected by nutrient–water ratio	Rheology-adjusted oyster mushroom pastes printable with decent structural fidelity; good shape retention at optimized moisture content	[59]
*P. eryngii* protein paste (20% solids)	Extrusion mode: Syringe Nozzle/Speed/Pressure: Not reported Temperature: Room temp	Good extrudability and shape retention at 20% PEP; formulation suitable for baked snack applications	[38]
Button mushroom powder + wheat flour (snack)	Nozzle: Ø 1.28 mm Printing speed: 800 mm/min Pressure: 4 bar Post-processing: microwave (800 W, 10 min)	Accurate constructs (~78% fidelity); 5.4% shrinkage after cooking; savory version rated best in sensory analysis	[10]
Mushroom by-product flour + potato flour + olive oil	Formulation: 2–6% mushroom flour, 5% olive oil, and 8–14% solids Extrusion type: Syringe (assumed) Nozzle/Speed/Pressure: Not reported	Mushroom flour reduced viscosity, olive oil increased it; high solid inks (14%) gave best shape and extrusion consistency	[60]

**Table 3:** Macronutrient profile of *P. ostreatus* (per 100 g dry weight).

Macronutrient	Content (Per 100 g dw)	Nutritional Significance	Ref.
Carbohydrates	43.42	Primary energy component	[68,71]
Crude Fiber	23.63	Contributes to digestive health	[68,71]
Protein	17.06	Complete amino acid profile that meets adult requirements	[68,71]
Ash Content	8.22	Indicates rich mineral presence	[68,71]
Lipids	1.21	Aligns with low-fat dietary recommendations	[68,71]

## Data Availability

All data are within the manuscript.

## Abbreviations

The following abbreviations are used in this manuscript:

*P. ostreatus*	*Pleurotus ostreatus*
3DFP	3D food printing
µCT	Micro-computed tomography
LDL	Low-density lipoprotein
EFSA	European Food Safety Authority
FDA	Food and Drug Administration
HACCP	Hazard Analysis and Critical Control Points
